# The effect of anthropogenic pressure shown by microbiological and chemical water quality indicators on the main rivers of Podhale, southern Poland

**DOI:** 10.1007/s11356-017-8826-7

**Published:** 2017-04-03

**Authors:** Anna Lenart-Boroń, Anna Wolanin, Ewelina Jelonkiewicz, Mirosław Żelazny

**Affiliations:** 1 Department of Microbiology, University of Agriculture in Cracow, Mickiewicza Ave. 24/28, 30-059 Cracow, Poland; 2 Department of Hydrology, Institute of Geography and Spatial Management, Jagiellonian University in Cracow, Gronostajowa 7, 30-387 Cracow, Poland

**Keywords:** Coliforms, *E. coli*, Water quality, Podhale, Anthropopressure

## Abstract

This study was aimed to determine the spatial variation in anthropogenic pressure exerted on surface water in the Podhale region (southern Poland), which is one of the most popular tourist destinations in Poland. The assessment was based on the dynamics and relationships between microbiological and chemical indicators of water quality throughout the major rivers of this region—Dunajec, Czarny Dunajec, Biały Dunajec, and Białka. Another aim was to assess the effect of land use on the quality of water in the studied rivers. The study was conducted over 1 year at 21 sampling sites distributed from the uppermost sections of rivers in the Tatra National Park through main tourist resorts until mouths of the considered rivers to the Czorsztyńskie Lake. Microbiological analysis comprised the prevalence of total and fecal types of coliforms and *Escherichia coli*, mesophilic, and psychrophilic bacteria. Chemical assays determined the concentrations of Na^+^, K^+^, NH_4_^+^, Cl^−^, NO_3_^−^, and PO_4_^3−^. Temperature, electrical conductivity, and pH were measured onsite. It was demonstrated that there is a significant relationship between the predominant types of land use within individual catchments, which results in evident differences in the pollution of waters between the catchments. The results showed that increased share of built-up areas and arable land results in significant deterioration of water quality. Thus, waters of Czarny Dunajec were the cleanest, while Biały Dunajec was the most heavily contaminated. Also, spatial diversity in water quality was shown—the cleanest waters were sampled in the Tatra National Park and the pollution increased with the course of rivers. Point sources of pollution such as effluents from treatment plants or discharge of untreated sewage from households proved to be more important than non-point sources, such as surface runoff. Moreover, the important role of the Czorsztyńskie Lake in the purification of water was demonstrated.

## Introduction

The quality of water is one of the factors affecting health and safety of its consumers, as well as the suitability for its use in various economic aspects, such as plant and animal production, development in both production and non-production sectors and the condition of natural environment. Water resources of Poland are relatively low and constitute only 3% of the European total amount of water (Eurostat 2011). However, Poland’s water consumption is also one of the lowest in Europe, therefore not the amount of available water, but ensuring the best possible quality of water resources becomes more and more important, and at the same time challenging (Myszograj and Sadecka 2012). Water environment is increasingly threatened by people, due to the growth of population size, with its consequences in the form of intensification of agriculture and various forms of industry (Páll et al. 2013). This is coupled with a growing number of households located throughout catchments, together with limited numbers and efficiencies of sewerage systems, particularly in rural areas (Lenart-Boroń et al. 2016). Contamination of water can be the result of domestic activity, industry, and tourism. These types of anthropogenic pressure are related with the occurrence of point sources, such as discharge of treated and untreated sewage from treatment plants and households, livestock farms, and non-point sources such as urban and agricultural runoff or water birds (Kirschner et al. 2009). The quality of surface water also depends on the amount and type of discharged pollutants, as well as the susceptibility of water to degradation and its potential of self-purification (Ostroumov 2006).

Podhale is a cultural region that covers southernmost areas of Poland and includes catchments of two major rivers—Białka and Dunajec with their tributaries which flow into the retention reservoir—Czorsztyńskie Lake. The Polish part of the Dunajec catchment covers more than 3500 km^2^, while the catchment of Białka covers an area of 730 km^2^. Both rivers and the Czorsztyńskie Lake are situated in areas which are considered very clean and therefore having numerous health resorts. This, however, caused recent increase of touristic popularity of the Podhale region, resulting in intensive development of touristic infrastructure which had severe negative effects on the environmental quality, particularly the quality of water in rivers (Krąż 2012). This is due to the growing consumption of water resources, large amount of waste, but also due to emission of pollutants and discharge of sewage into rivers (Hełdak and Szczepanski 2011).

Due to a variety of ways of water usage in the considered region, monitoring and proper management of water quality is of great importance. Therefore, understanding of the extent and origin of contamination sources is in such cases crucial in planning of further management activities (Páll et al. 2013). Human and animal pathogens of enteric origin are considered important contaminants of the environment, including surface water. When assessing the sources and degree of water contamination with feces, a number of indicators are taken into consideration, among which *Escherichia coli* and coliforms are among most frequently applied ones in terms of microbiological contamination and the content of pathogenic microorganisms (Ashbolt et al. 2001). This is due to the fact that bacteria are convenient markers of pollution, as their population is proportional to the amount of feces delivered into environment (Páll et al. 2013).

The primary aim of this study was to determine the spatial variation in anthropogenic pressure exerted on surface water in the Podhale region, based on the dynamics and relationships between microbiological and chemical indicators of water quality throughout the major rivers of this region. The secondary aim was to assess the effect of land use and point sources of pollution on the quality of water in the studied rivers.

## Material and methods

### Study area and sampling

**Fig. 1:**
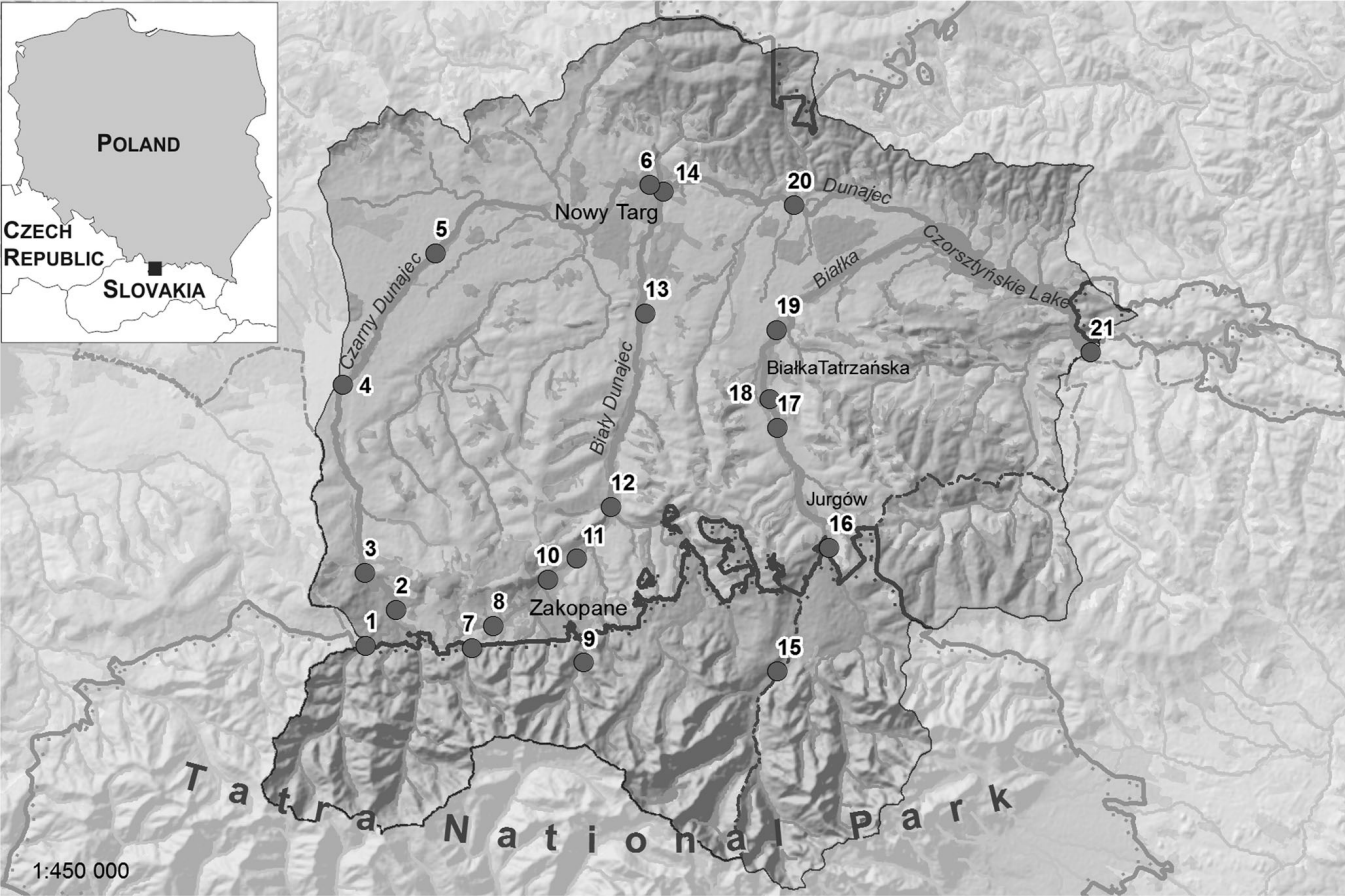
Study area and the location of the sampling sites

The samples of water were collected in 21 sites, located on major rivers of the Podhale region: Białka, Biały Dunajec, Czarny Dunajec, and Dunajec. The river Biały Dunajec joins with Czarny Dunajec near the town of Nowy Targ and further runs as the Dunajec river, which together with the Białka river receive water from the entire watershed and flow into the Czorsztyńskie Lake (Fig. 1). The Czorsztyńskie Lake is one of the largest mountain retention reservoirs, whose catchment covers 1147 km^2^. Through catchments of Białka and Dunajec, the reservoir receives water from both Polish and Slovakian Tatras, Subtatran Ditch, Spisko-Gubałowskie Foothills, Orawa-Nowy Targ Valley, Orava-Podhale Beskid, Gorce, Sądecki Beskid, and the Pieniny (Jaguś and Rzętała 2010). The location of the sampling points was chosen in a way that allows to observe the effect of broadly understood anthropogenic activity on the quality of the studied river waters and to demonstrate the most important pollution sources and mechanisms affecting the quality of surface water in the considered region.

The rivers Białka, Biały Dunajec, and Czarny Dunajec flow out of the Tatra National Park (TNP). The Tatra National Park is a protected area, anthropogenically untransformed and thus it is attractive tourist destination. Land cover of the TNP consists of five vegetation belts: lower montane belt (the Carpathian beech forest), upper montane belt (spruce forest), dwarf pine belt, alpine belt (high-altitude grassland), and subnival belt (bare rock and very sparse vegetation) (Mirek and Piękoś-Mirkowa 1992). Over the year the TNP is visited by more than 2.5 million people (Skawiński 2010). In the outskirts of the TNP, there are major tourist resorts, such as Zakopane, Białka Tatrzańska, or Kościelisko. That combined constitutes very high population density. In the lower areas of the examined catchments, the share of agricultural land, meadows, and pastures increases significantly (Table 1).

**Table 1:** Area and land use of the regions of the sampling sites

No. of sampling site	Name of sampling site	Characteristics of sampling site	Catchment	Area	Arable land	Built-up areas	Forests	Meadows and pastures
				km^2^	%			
1	Siwa	Siwa Polana clearing in TNP	Czarny Dunajec	34.22	0.0	0.0	96.6	3.4
2	TNP	Tatra National Park		78.79	0.0	0.0	95.5	4.5
3	Molkówka forest	Molkówka montane clearing		93.00	2.0	0.4	91.1	6.6
4	Koniówka	Koniówka village		133.13	11.3	1.7	68.6	18.4
5	Wróblówka	Wróblówka vilage		141.26	12.4	3.4	65.8	18.4
6	Czarny Dunajec Nowy Targ	River Czarny Dunajec in the town of Nowy Targ		434.70	22.3	4.4	33.7	39.6
7	Małołącki stream	Stream in the Western Tatra Mountains	Biały Dunajec	5.56	0.0	0.0	99.1	0.9
8	Upstream of the hospital	Zakopane town, upstream of a large local hospital		15.25	11.2	9.0	67.6	12.1
9	Bystra stream	Stream in the Western Tatra Mountains		14.60	0.1	0.0	97.7	2.2
10	Downstream of the hospital	Zakopane town, downstream of a large local hospital		53.87	5.4	14.6	70.0	10.0
11	Downstream of the SEWIK treatment plant	Zakopane town, downstream of the municipal STP		59.18	8.1	15.1	64.8	12.0
12	Poronin Bridge	Poronin town, downstream of the Poroniec tributary		164.19	10.6	8.6	61.8	19.0
13	Szaflary Bridge	Szaflary town, popular accommodation place for tourists		209.84	14.1	8.5	51.2	26.2
14	Biały Dunajec Nowy Targ	River Biały Dunajec in the town of Nowy Targ		226.25	15.2	9.1	48.8	26.8
15	Białka Łysa Polana	Border crossing with Slovakia, border of the TNP	Białka	63.76	0.0	0.0	62.3	37.7
16	Jurgów intake	Water intake for snowing of ski slopes in Jurgów village		94.20	0.4	0.0	87.3	12.3
17	Upstream of the STP	Czarna Góra village, upstream of the municipal treatment plant		188.38	8.4	0.6	65.3	25.8
18	Białka intake	Water intake for snowing of ski slopes in Białka village		190.15	8.6	0.7	65.0	25.7
19	Białka Trybsz	Bridge in the Trybsz village, a popular accommodation place for tourists		200.59	9.1	1.0	63.2	26.7
20	Dunajec Łopuszna	Bridge in Łopuszna village, popular accommodation place for tourists, upstream of the Czorsztyńskie Lake	Dunajec	777.64	21.1	5.9	39.0	34.0
21	Dunajec downstream of the Czorsztyńskie Lake	Niedzica village, downstream of the dam on the Czorsztyńskie Lake		1270.49	22.2	4.3	44.7	28.8

Size of catchments was calculated using GIS system and data concerning the land use were obtained from the system CORINE Land Cover 2012

Water samples were collected over a period of 1 year—from December 2014 to December 2015 in 12 sampling campaigns. For microbiological analyses, the samples were collected into 1000 ml sterile polypropylene bottles and for chemical analyses—into 500 ml polyethylene bottles. Measurements of electrical conductivity (EC_25°C_) and temperature of water were conducted onsite during sampling, using a Pro 2030 Multimeter handheld (YSI, USA).

### Laboratory analyses

The membrane filtration method was applied to determine the number of total coliforms and thermotolerant (fecal) coliforms (purple red colonies with metallic sheen on Endo agar, incubation for 48 h, at 37 and 44 °C, respectively), as well as total and thermotolerant *E. coli* (blue-green colonies on TBX agar, incubation for 48 h at 37 and 44 °C, respectively). The number of mesophilic and psychrophilic bacteria was determined using serial dilutions method (trypticase soya agar, 37 °C for 48 h and 4 °C for 72 h, respectively).

After filtration of water with 0.45 μm syringe filter, the concentration of the following ions in water: Na^+^, K^+^, NH_4_^+^, Cl^−^, NO_3_^−^, and PO_4_^3−^ was measured with DIONEX ICS-2000 chromatographic system.

Analysis of variance (ANOVA) and post-hoc Scheffe test for *p* = 0.95 were applied in order to verify whether there are significant differences in the values of microbiological and physico-chemical water quality indicators between the studied locations. The effect of the catchment size and land use on the quality of water in the studied catchments was determined using the Spearman correlation coefficient. Student’s *t* test was used to analyze the significance of differences in the values of microbiological and physico-chemical indicators between waters flowing into and out of the Czorsztyńskie Lake.

## Results and discussion

Table 2 presents mean values of microbiological and physico-chemical indicators of water quality for individual studied catchments. Mean water pH was 7.4–7.7 and conductivity (EC_25°C_) ranged from ~200 to ~330 μS/l. The concentrations of nitrogen and phosphorus compounds were high only in the waters from the Biały Dunajec catchment. On the other hand, the mean values of microbiological indicators were very diverse, particularly in the case of both total and fecal types of coliforms and *E. coli*. For instance, the mean concentrations of fecal *E. coli* ranged from 158 CFU/100 ml (Czarny Dunajec) to 11,800 CFU/100 ml (Biały Dunajec) and the range was even greater in the case of total coliforms, i.e., from 380 CFU/100 ml in the Czarny Dunajec catchment to 29,800 CFU/100 ml in the catchment of Biały Dunajec. This indicates that the mean values of almost all parameters, both microbiological and physico-chemical ones, were the highest in the Biały Dunajec catchment. On the other hand, the smallest amounts of most bacterial indicators were recorded in the catchment of Czarny Dunajec, while for physico-chemical indicators such situation was observed in the catchment of the Białka river.

**Table 2:** Mean values of microbiological and physico-chemical indicators of water quality in the examined catchments

Parameter	Unit	Catchment		
		Białka	Czarny Dunajec	Biały Dunajec
Fecal *E. coli*	CFU/100 ml	1000	158	11,800
Fecal coliforms		1750	271	6440
Total *E. coli*		1150	228	12,670
Total coliforms		2230	379	29,800
Mesophilic bacteria	CFU/ml	1110	1610	6840
Psychrophilic bacteria		3510	3520	30,600
pH	–	7.5	7.7	7.4
EC	μS/cm	201.6	254.1	328.4
Na	mg l^−1^	2.65	3.80	11.78
K		0.65	0.88	1.69
NH_4_		0.0294	0.0153	0.0281
Cl		3.21	4.19	16.60
NO_3_		2.78	2.95	6.19
PO_4_		0.0169	0.0040	0.1728

The observed differences in the values of water quality indicators result from differences in the type of land use and intensity of touristic activity within the examined catchments (Smoroń and Twardy 2003; Lenart-Boroń et al. 2016). This is due to the fact that the main source of water contamination in the considered area includes either effluents from treatment plants, illegal discharge of untreated sewage from households or surface runoff carrying bacteria from natural fertilizers, e.g., manure (Lenart-Boroń et al. 2016; Mazurkiewicz-Boroń 2002). Smoroń and Twardy (2003) in their study on the effect of variable intensity of tourist movement on the quality of waters in Czarny and Biały Dunajec also reported that waters of Biały Dunajec were characterized by significantly higher contamination with the concentration of fecal coliforms eight times higher than in Czarny Dunajec.

### Assessment of water quality

Fecal pollution of the analyzed water samples and classes of microbiological quality of water were determined based on the Bathing Water Directive (Directive 76/160/EEC 2006). Out of the 21 analyzed sites, 8 were characterized by excellent quality (Table 3). In most cases, these are waters flowing out of the Tatra National Park, characterized by low or no contamination with *E. coli*. The best microbiological quality was observed for waters of the Czarny Dunajec catchment, where poor quality of water was observed only in one sampling site. On the other hand, the worst microbiological quality was recorded in the catchment of Biały Dunajec, where except from sites located in the Tatra National Park, the values of microbiological indicators exceed the limit values several times. The results of physico-chemical analyses were compared with values given in the Journal of Laws 1482, 2014, Regulation of the Minister of Environment on the classification of surface water bodies and environmental quality standards for priority substances.

**Table 3:** Classes of microbiological and physico-chemical quality in the sampling sites according to the Bathing Water Directive and J. of Laws 1482

Sampling site (no.)	Catchment	Microbiological class	Physico-chemical class
1	Czarny Dunajec	I—excellent quality	I—very good ecological status
2		I—excellent quality	I—very good ecological status
3		I—excellent quality	I—very good ecological status
4		I—excellent quality	I—very good ecological status
5		II—good quality	I—very good ecological status
6		IV—poor quality	I—very good ecological status
7	Biały Dunajec	I—excellent quality	I—very good ecological status
8		IV—poor quality	I—very good ecological status
9		I—excellent quality	I—very good ecological status
10		IV—poor quality	II—good ecological status
11		IV—poor quality	III—below good ecological status
12		IV—poor quality	I—very good ecological status
13		IV—poor quality	II—good ecological status
14		IV—poor quality	I—very good ecological status
15	Białka	I—excellent quality	I—very good ecological status
16		I—excellent quality	I—very good ecological status
17		IV—poor quality	I—very good ecological status
18		IV—poor quality	I—very good ecological status
19		IV—poor quality	I—very good ecological status
20	Dunajec	IV—poor quality	I—very good ecological status
21		IV—poor quality	I—very good ecological status

The situation in the examined catchments is significantly better in terms of physico-chemical parameters. Waters in the catchments of Czarny Dunajec and Białka are characterized by the best quality, i.e., I—very good ecological status (Table 3). Also, due to increased values of NO_3_ and PO_4_ waters were qualified into II class of quality in two sampling sites (downstream of the hospital and Szaflary—no. 10 and 13) of the catchment of Biały Dunajec. In contrast, in the sampling site downstream of the SEWIK treatment plant (no. 11), the quality of water is poor, due to high concentrations of PO_4_. Smoroń and Twardy (2006) suggest that the main causes of deterioration of water quality in the Biały Dunajec catchment (i.e., increasing concentrations of nitrogen and phosphorus compounds) are high population density, intensive tourism, and inadequate water and sewage management, especially ineffective operation of sewage treatment plants during the highest tourist seasons.

On the other hand, in the catchment of the Białka river, Lenart-Boroń et al. (2016) reported significant deterioration of water quality, mainly due to increased tourist traffic in recent years, resulting in growing number of illegal sewage discharge sites throughout the river and insufficient efficiency of the local treatment plant. Another important source of water contamination in the catchment of Białka is related to natural processes, such as surface runoff, snowmelt water, and soil leaching. Earlier study concerning the quality of water in the Upper Dunajec watershed, conducted by Szalińska and Dominik (2006) indicated that most significant fecal pollution occurred in waters of the Dunajec river, whereas sanitary quality of Białka (with sampling site located in Trybsz) was the best. This indicates that the observed contamination of water may have indeed increased in recent years and that it will continue to deteriorate. Szalińska and Dominik (2006) also concluded that contamination of water in the considered region results mostly from intensive use of watershed for sheep and cattle grazing along with discharge of untreated sewage. Improvement of capacity of sewage treatment plants in the region and the construction of new ones allows to expect that the contamination of water will either remain on the constant level or might improve.

### Spatial diversity of water quality

**Fig. 2:**
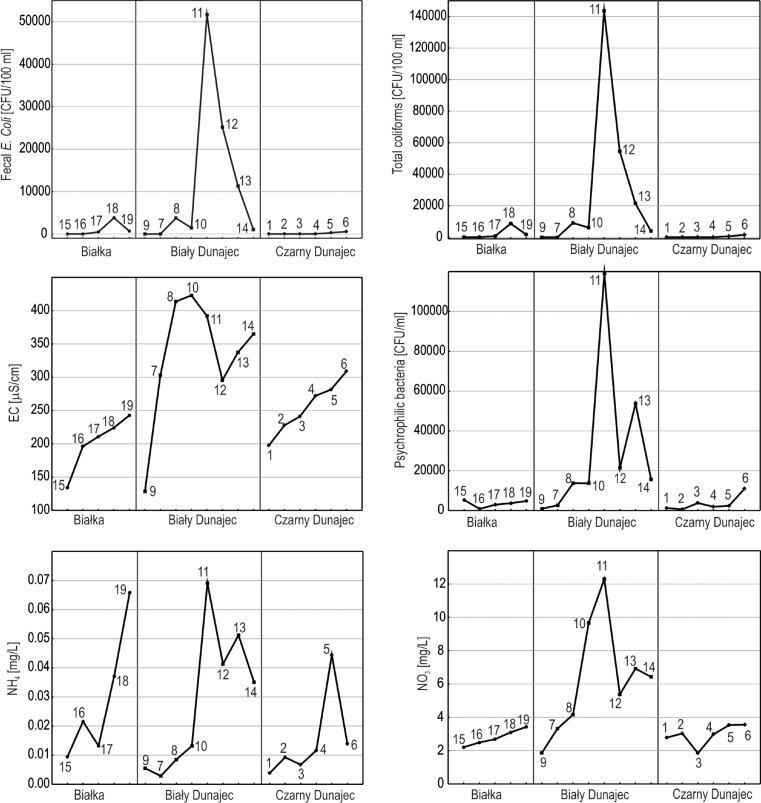
Changes in microbiological and physico-chemical parameters of water quality along the course of rivers Białka, Biały Dunajec, and Czarny Dunajec. *Numbers 1–19* correspond to the numbers of the sampling sites

Spatial diversity of microbiological and physico-chemical indicator values in the studied catchments is evident (Fig. 2). In the case of the Czarny Dunajec catchment, the lowest mean concentrations of Na^+^, K^−^, NH_4_^−^, Cl^−^, NO_3_^−^, and PO_4_^3−^ ions as well as the number of bacteria were observed in the Tatra National Park. The values of the studied indicators increase with the course of the river. The highest values of most parameters were observed in Nowy Targ (no. 6). In the catchment of the Białka river the lowest values of the tested parameters also occur in the Tatra National Park (sample Białka Łysa Polana, no. 15). On the other hand, the concentrations of microbiological indicators and PO_4_^3−^ are the highest at the sampling site intake (no. 18), situated downstream of the discharge from the sewage treatment plant, while in the further course of the river, values of these parameters decrease. EC_25°C_ and the concentrations of Na^+^, K^−^, NH_4_^−^, Cl^−^, and NO_3_^−^ in waters of Białka increase along its course to reach the highest values in the sampling site Trybsz (no. 19). Also, in the catchment of Biały Dunajec, the lowest values of microbiological and physico-chemical indicators were recorded in the Tatra National Park (samples Bystra—no. 9 and Małołącki stream—no. 7). Downstream of the discharge from the sewage treatment plant in Zakopane (sampling site: downstream of the SEWIK, no. 11) there is a rapid increase in the number of all bacteria, EC_25°C_ and Na^+^, K^−^, NH_4_^−^, Cl^−^, NO_3_^−^, and PO_4_^3−^. At the site Poronin Bridge (no. 12), the values of the examined microbiological and physico-chemical parameters drop again and then in the further course of the river either increase slightly or remain at the same level.

It is evident that the highest increase in the number of bacteria and nutrient concentrations in waters occurs downstream of discharge sites from the sewage treatment plants. This is clearly noticeable in the catchments of Biały Dunajec (sampling site downstream of the SEWIK, no. 11) and Białka (intake, no. 18). These results are consistent with the observations of, e.g., Servais et al. 2007, who found that point sources of pollution, particularly of discharge sites from treatment plants, have much higher impact on the deterioration of microbiological quality of water than the presence of non-point sources. Also, studies conducted earlier in the considered region demonstrated that sources of sewage discharge into rivers Białka (Lenart-Boroń et al. 2016) and Dunajec (Szalińska and Dominik 2006) have most significant impact on the quality of water in their catchments. According to Whitehead and Lack (1982), the principal effects of such point sources of pollutants include the risk of disease transmission, loss of oxygen resources, water eutrophication, and finally—loss of esthetic values. Apart from being major sources of water supply, rivers act as principal disposal pathways for waste materials. With increasing development of economy, the variety of waste types increases and the problem of water quality becomes more difficult and demanding than the quantity of water. Therefore, it becomes essential to properly manage river systems and to maintain adequate water quality (Whitehead and Lack 1982).

In the catchments of Białka and Biały Dunajec, the values of microbiological indicators drop at the sampling sites located further downstream of these major point sources of pollution. Even though the concentrations of coliforms and *E. coli* are still very high, they are several times lower than the maximum ones recorded directly by the discharge sites (i.e., no. 11 and 18 in the catchments of Biały Dunajec and Białka, respectively). Such decrease can be explained by the processes of self-purification of waters, among which dilution of contaminants is the simplest and most probable one in the studied cases (Jaroszewicz 2007).

Based on the ANOVA test, it was examined whether there are statistically significant differences in the values of the tested water quality indicators in waters from different sampling sites (specific data not shown). In the catchment of the Białka river, the differences were statistically significant for most parameters with exception of psychrophilic bacteria, pH, NH_4_^+^, and NO_3_^−^. The sampling sites Łysa Polana (no. 15) and Jurgów (no. 16) differed significantly from the sites Trybsz (no.19) and intake (no. 18). In the case of potassium ions, the differences are significant between almost all sampling sites—these concentrations only at the site intake (no. 18) do not differ from the values observed in the sites Trybsz (no. 19) and upstream of the STP (no. 17).

No significant differences were found between the sampling sites of the Czarny Dunajec catchment for fecal *E. coli*, psychrophilic bacteria, pH, NH_4_^+^, and PO_4_^3−^. For other parameters, statistically significant differences were found between the sites situated in the Tatra National Park (no. 1, no. 2) and Czarny Dunajec in Nowy Targ (no. 6). The concentration of potassium ions differs significantly between all sampling sites, except from Siwa-TPN (no. 1) and Wróblówka (no. 5)-Koniówka (no. 4), while for the concentrations of NO_3_^−^, significant differences were found between the sites Molkówka (no. 3) and Wróblówka (no. 5), and Nowy Targ (no. 6).

The differences between sampling sites of the Biały Dunajec catchment were statistically significant with only exception of NH_4_^+^ and NO_3_^−^ ions. Sites Bystra (no. 9) and Małołącki (no. 7) differ significantly from other sites in terms of the prevalence of fecal *E. coli*, fecal coliforms, total *E. coli*, total coliforms, mesophilic, and psychrophilic bacteria, and also in the concentration of Na^+^, K^+^, Cl^−^, and PO_4_^3−^ ions. Also, the site downstream of SEWIK (no. 11) differs significantly from all other sites, except for Szaflary (no. 13) and Poronin (no. 12) in terms of fecal *E. coli*, fecal coliforms, total *E. coli*, and total coliforms. Water pH differs significantly between the sites downstream of the SEWIK (no. 11) and Małołącki stream (no. 7).

The differences in the water quality indicators, supported with the analysis of variance demonstrate the anthropogenic pressure on the waters along the course of the examined rivers. Values of the tested parameters in waters of rivers flowing out of the Tatra National Park differ significantly from those observed in waters in areas more transformed by agriculture and sewage discharge sites. However, the biggest threat to the quality of surface waters identified in previous studies is related to high population density and the effect of intensive tourism in the catchment of the Biały Dunajec river (Smoroń et al. 2007). The quality of waters in the catchment of Białka is threatened mostly by intensive winter tourism, resulting in increased population density and thus increased production of sewage (Lenart-Boroń et al. 2016). On the other hand, low intensity of agricultural production results in much smaller impact on water quality deterioration (Smoroń and Kowalczyk 2008), which is clearly visible in the Czarny Dunajec catchment.

### The impact of land use on water quality

Table 4 shows coefficients of correlation between water quality parameters and the size of catchments and land use.

**Table 4:** The coefficients of correlation between the size of catchments (km^2^), land use (%), and water quality parameters

Catchment	Land use	Fecal *E. coli*	Fecal coliforms	Total *E. coli*	Total coliforms	Mesoph. bacteria	Psychroph. bacteria	EC	Na	K	NH_4_	Cl	NO_3_	PO_4_
Biały Dunajec	Area	0.16	0.15	0.12	0.11	0.11	0.10	*−0.45*	*−0.43*	*−0.22*	*−0.38*	*−0.41*	*−0.37*	*−0.23*
	Arable land	0.10	0.11	0.08	0.08	0.12	0.07	−0.15	−0.15	0.02	−0.15	−0.14	*−0.23*	−0.14
	Built-up areas	−0.06	−0.06	−0.04	−0.03	−0.04	−0.02	*0.62*	*0.74*	*0.56*	*0.41*	*0.72*	*0.67*	*0.45*
	Forests	−0.10	−0.11	−0.08	−0.09	−0.12	−0.07	0.09	0.05	−0.10	0.10	0.04	0.12	0.07
	Meadows and pastures	0.12	0.12	0.09	0.09	0.13	0.08	*−0.24*	*−0.21*	−0.03	−0.19	−0.21	−*0.26*	−0.17
Czarny Dunajec	Area	0.24	0.25	0.24	0.25	0.25	0.17	*0.57*	*0.53*	*0.80*	*0.30*	*0.42*	0.10	0.29
	Arable land	0.27	0.29	0.28	0.29	0.29	0.19	*0.52*	*0.43*	*0.85*	*0.32*	0.30	0.19	*0.32*
	Built-up areas	*0.35*	*0.38*	*0.37*	*0.38*	*0.38*	0.19	*0.49*	*0.40*	*0.83*	*0.38*	*0.31*	0.23	*0.38*
	Forests	−0.27	−0.29	−0.28	−0.29	−0.29	−0.19	*−0.53*	*−0.43*	*−0.85*	*−0.32*	*−0.31*	−0.19	*−0.32*
	Meadows and pastures	0.25	0.27	0.26	0.27	0.27	0.18	*0.53*	*0.43*	*0.84*	*0.31*	*0.31*	0.18	*0.31*
Białka	Area	0.24	0.25	0.27	0.20	*0.40*	−0.06	*0.46*	*0.62*	*0.73*	0.20	*0.62*	*0.39*	*0.38*
	Arable land	0.25	0.26	0.28	0.20	*0.40*	−0.03	*0.42*	*0.61*	*0.73*	0.20	*0.60*	*0.37*	*0.39*
	Built-up areas	0.20	0.21	0.23	0.16	*0.44*	0.04	*0.43*	*0.69*	*0.81*	0.26	*0.66*	*0.41*	*0.42*
	Forests	−0.13	−0.13	−0.14	−0.10	−0.22	−0.23	0.05	−0.26	*−0.34*	−0.07	−0.18	−0.10	−0.23
	Meadows and pastures	0.01	0.01	0.01	0.00	0.03	0.29	*−0.30*	−0.04	−0.02	−0.03	−0.13	−0.09	0.05

Values in italics show statistically significant correlations (*p* = 0.05)

In the catchment of Czarny Dunajec, there is a statistically significant increase in the number of fecal *E. coli*, fecal coliforms, total *E. coli*, total coliforms, and mesophilic bacteria with increasing surface of built-up areas. There is also a positive, statistically significant relationship between EC_25°C_, concentrations of Na^+^, K^+^, NH_4_^+^, PO_4_^3−^, and the catchment size, size of arable land, built-up areas, meadows and pastures. On the other hand, values of these parameters are negatively correlated with size of areas covered by forests. In turn, there is a statistically significant positive correlation between concentration of Cl^−^ and the size of the catchment as well as the size of built-up areas, meadows and pastures, while the correlation is negative for the size of areas covered with forests.

In the catchment of Białka, there is a statistically significant positive correlation between the content of mesophilic bacteria and the size of the catchment, arable land and built-up areas. On the other hand, the concentrations of Na^+^, Cl^−^, NO_3_^−^, and PO_4_^3−^ are positively correlated with the size of the catchment, arable lands and built-up areas. Water conductivity (EC_25°C_) is positively correlated with the catchment size, arable lands, built-up areas and negatively—with size of meadows and pastures. The concentration of potassium is positively correlated with the catchment size, arable lands and built-up areas, while negatively—with forests.

These relationships are entirely different in the catchment of Biały Dunajec. There are no significant relationships between bacteriological parameters of water quality and the catchment size or the land use. The concentrations of K^+^, NH_4_^+^, Cl^−^, and PO_4_^3−^ are negatively correlated with the catchment size and positively with the size of built-up areas. The Na^+^ concentration and EC_25°C_ are correlated negatively with the catchment size and meadows and pastures. The concentration of nitrates is positively correlated with the size of built-up areas.

Based on the obtained correlations it can be concluded that physico-chemical parameters of water quality are more sensitive to the effect of the size of catchments and land use than bacteriological water quality indicators. The increase in the share of built-up areas in catchments undoubtedly results in deterioration of water quality. This is particularly evident in the case of increased concentrations of nitrogen and phosphorus compounds. Increased concentration of NO_3_^−^ in water coupled with increasing share of built-up areas was also observed by Ahearn et al. (2005). On the other hand, in the catchment of Czarny Dunajec, it was observed that the concentration of bacteria in water increased with greater share of built-up areas. Also, St Laurent and Mazumder (2012) observed a positive correlation between concentrations of fecal coliforms in water and urban land use type. However, they also emphasize that the fecal contamination is higher in areas used for agricultural purposes. Also, Tong and Chen (2002) found that there is a relationship between water quality parameters and land use. It was particularly evident that there is a strong positive relationship between total nitrogen, total phosphorus, and fecal coliforms with the share of commercial, residential, and agricultural lands. In contrast, this correlation was negative for forest-covered areas.

### The role of the Czorsztyńskie Lake

Table 5 shows mean values of microbiological indicators of water quality and physico-chemical parameters of water flowing into and out of the Czorsztyńskie Lake, demonstrating that inflowing water is characterized by significantly higher values of microbiological indicators than water flowing out of the Lake. The number of bacteria in inflowing water (sample Dunajec Łopuszna—no. 20) is several times higher than in effluent from the reservoir (sample Dunajec downstream of the Czorsztyńskie Lake—no. 21). This difference is particularly evident for total coliforms. Water flowing out of the Lake is characterized by reduced pH and the concentration of Na^+^, NH_4_^+^, Cl^−^, and PO_4_^3−^ ions compared with inflowing water. In contrast, values of EC_25°C_ and potassium ion concentration increase, while the concentration of NO_3_^−^ remains the same in both groups of samples. Based on the Student’s *t* test, it was concluded that waters flowing out of the Czorsztyńskie Lake differ significantly from those flowing into the lake in terms of bacteriological indicators of water quality and the concentrations of K^+^, NH_4_^+^, and PO_4_^3−^ ions. Similar observations were described by Szalińska and Dominik (2006), who reported that water flowing out of the Czorsztyńskie Lake is characterized by significantly lower concentrations of nitrogen and phosphorus compounds.

**Table 5:** Mean values of microbiological and physico-chemical indicators of water quality in water flowing into and out of the Czorsztyńskie Lake

Parameter	Unit	Water flowing into the lake	Water flowing out of the lake
Fecal *E. coli*	CFU/100 ml	930	220
Fecal coliforms		1600	270
Total *E. coli*		1900	240
Total coliforms		2900	300
Mesophilic bacteria	CFU/ml	2000	840
Psychrophilic bacteria		5100	1090
pH	–	7.6	7.4
EC_25°C_	μS/cm	299.5	313.7
Na^+^	mg l^−1^	8.85	8.75
K^+^		1.68	2.20
NH_4_^+^		0.034	0.013
Cl^−^		10.94	10.43
NO_3_^−^		4.36	4.36
PO_4_^3−^		0.039	0.015

Mean annual concentration of nitrogen compounds (NH_4_^+^ and NO_3_^−^) and phosphates (PO_4_^3−^) in waters of rivers flowing into the Czorsztyńskie Lake reaches the following values: NH_4_^+^—0.034 mg l^−1^, NO_3_^−^—4.36 mg l^−1^, and PO_4_^3−^—0.039 mg l^−1^. Concentrations of those nutrients in waters flowing into the Lake are not high, however, on average ~667 t N-NH_4_ and N-NO_3_, and ~8.5 t P-PO_4_ flows into the lake over the entire year, whereas the values of nutrients flowing out of the Lake are as follows: ~650 t N-NH_4_ and N-NO_3_ and ~3 t P-PO_4_. Therefore, ~17 t/year N-NH_4_ and N-NO_3_ and ~5.5 t/year P-PO_4_ remains in the reservoir. Phosphorus compounds are probably stored in the reservoir sediments as a result of adsorption and sedimentation processes (Stutter and Lumsdon 2008; Tye et al. 2016). Similar situation can be observed in the case of nitrogen compounds, which are also deposited in the reservoir sediments (Kemp and Mudrochova 1972). Retention of nitrogen in the reservoir is also the result of its accumulation in biomass and denitrification (Canham et al. 2012).

Having regard to the fact that the Czorsztyńskie Lake acts not only as a storage reservoir, but is also used for recreational purposes—mostly sailing, windsurfing, bathing, and fishing (Jaguś and Rzętała 2010), it is important to recognize and understand issues related to its contamination, including the concentration and fate of waterborne pathogens. Water storage has been proved to be among factors causing decrease in pathogen concentration in surface waters (Brookes et al. 2004). Distribution and transport of microorganisms within a reservoir is a function of pathogen load in inflowing water, sedimentation, entrainment, and resuspension of pathogens from settled particles by turbulence in the benthic boundary layer (Walker and Stedinger 1999). Viability of pathogens within reservoirs is mainly affected by temperature and UV light, water pH, and—among biological parameters—predation by protozoa or invertebrates (Simek et al. 2001). The mentioned factors may have significantly affected the concentration of bacterial indicators of water quality in the Czorsztyńskie Lake, particularly the number of coliforms and *E. coli*. These are enteric microorganisms, which have evolved to exist in relatively stable environment, therefore their exposure to unfavorable conditions which can be encountered in rivers and water reservoirs can be a significantly limiting factor. Studies by Murphy et al. (2010) demonstrated that populations of *E. coli* can be inactivated within a warm upper layer of a shallow reservoir, but they can also survive for long periods of time within a cool, oxygen depleted environments in lower layers of these same systems. The range of factors affecting microbial fate and transport in a reservoir is wide and complex, and their detailed analysis is beyond the scope of this paper.

## Conclusions

The conducted study showed that the most significant sources of water contamination in the entire Podhale region include point sources such as effluents from sewage treatment plants or illegal discharge from households and—to a lesser extent—surface runoff carrying bacteria. The four considered catchments, situated in Podhale vary in terms of the predominant type of land use and tourism intensity, hence the observed differences in the concentrations of the examined water quality indicators. The catchment size and land use have more significant impact on physico-chemical parameters than on bacterial indicators of water quality.

Among the examined catchments, waters of Biały Dunajec and Dunajec are characterized by the worst quality, while the cleanest waters are observed in the Czarny Dunajec river. Microbiological parameters indicate significant contamination of waters in more sampling sites than physico-chemical parameters do. Diversity in the concentrations of water quality indicators was evident throughout all studied rivers with the lowest values observed in the uppermost sections of rivers, located in the Tatra National Park. The highest contamination was observed in urbanized areas, with particularly clear impact of effluents from sewage treatment plants.

The results obtained in this study also demonstrated a significant role of the Czorsztyńskie Lake in purification of surface water. Almost all parameters analyzed in this study were improved in water flowing out of the reservoir compared to inflowing water.

Having regard to the fact that in Poland, the issues related to water quality are more demanding than its scarcity, proper management of river systems becomes essential. Among important aspects of such management is careful selection of sampling sites when designing a monitoring program, in order to properly understand the processes and mechanisms affecting the quality of water within a catchment.

## References

[CR1] Ahearn DS, Sheibley RW, Dahlgren RA, Anderson M, Johnson J, Tate KW (2005). Land use and land cover influence on water quality in the last free-flowing river draining the western Sierra Nevada, California. J Hydrol.

[CR2] Ashbolt NJ, Grabow WO, Snozzi M, Fewtrell F, Bartram J (2001). Indicators of microbial water quality. Water quality—guidelines, standards and health assessment of risk and risk management for water-related infectious disease.

[CR3] Brookes JD, Antenucci J, Hipsey M, Burch MD, Ashbolt NJ, Ferguson C (2004). Fate and transport of pathogens in lakes and reservoirs. Environ Int.

[CR4] Canham CD, Pace ML, Weathers KC, McNeil EW, Bedford BL, Murphy L, Quinn S (2012). Nitrogen deposition and lake nitrogen concentrations: a regional analysis of terrestrial controls and aquatic linkages. Ecosphere.

[CR5] Directive 2006/7/EC of the European Parliament and of the Council of 15 February 2006 concerning the management of bathing water quality and repealing Directive 76/160/EEC (http://ec.europa.eu/environment/water/water-bathing/index_en.html)

[CR6] Eurostat (2011). Statistical books, Europe in figures, Eurostat yearbook.

[CR7] Hełdak M, Szczepanski J (2011). Changes of the landscape village of Bialka Tatrzanska from the development tourism. Infrastructure and Ecology of Rural Areas.

[CR8] Jaguś A, Rzętała M (2010). The Czorsztyn and Sromowce Reservoirs—location, characteristics and nomenclature. Pieniny–Dam–Changes–The Pieniny Monographs.

[CR9] Jaroszewicz A (2007). Self-purification process in river ecosystems. Słupsk Biological Papers.

[CR10] Journal of Laws of the Republic of Poland, item 1482. Regulation of the Minister of Environment of 22 October 2014 on the classification status of surface water and environmental quality standards for priority substances

[CR11] Kemp ALW, Mudrochova A (1972). Distribution and forms of nitrogen in a Lake Ontario sediment core. Limnol Oceanogr.

[CR12] Kirschner AKT, Kavkaa GG, Velimirov B, Mach RL, Sommer R, Farnleitner AH (2009). Microbiological water quality along the Danube River: integrating data from two whole-river surveys and a transnational monitoring network. Water Res.

[CR13] Krąż P (2012). Anthropogenic hazards to the Białka Valley natural environment. Geographical Papers.

[CR14] Lenart-Boroń A, Wolanin A, Jelonkiewicz Ł, Chmielewska-Błotnicka D, Żelazny M (2016). Spatiotemporal variability in microbiological water quality of the Białka river and its relation to the selected physicochemical parameters of water. Water Air Soil Poll.

[CR15] Mazurkiewicz-Boroń G (2002). Factors affecting eutrophication of the submountain dam reservoirs. Suppl Acta Hydrobiol.

[CR16] Mirek Z, Piękoś-Mirkowa H (1992). Flora and vegetation of the Polish Tatra Mountains. Mt Res Dev.

[CR17] Murphy M, Jamieson R, Gordon R, Stratton GW, Madani A (2010). Inactivation of *Escherichia coli* during storage of irrigation water in agricultural reservoirs. Can Water Resour J.

[CR18] Myszograj S, Sadecka Z (2012). Realization of National Programme of Municipal Wastewater Treatment and the quality of surface water in Poland. Environ Med.

[CR19] Ostroumov SA (2006). Biomachinery for maintaining water quality and natural water self-purification in marine and estuarine systems: elements of a qualitative theory. International Journal of Oceans and Oceanography.

[CR20] Páll E, Niculae M, Kiss T, Şandru CD, Spĭnu M (2013). Human impact on the microbiological water quality of the rivers. J Med Microbiol.

[CR21] Servais P, Garcia-Armisen T, George I, Billen G (2007). Fecal bacteria in the rivers of the Seine drainage network (France): sources, fate and modeling. Sci Total Environ.

[CR22] Simek K, Pernathaler J, Weinbauer M, Hornak K, Dolan JR, Nedoma J, Mašín M, Amann R (2001). Changes in the bacterial community composition and dynamics and viral mortality rates associated with enhanced flagellate grazing in a mesotrophic reservoir. Appl Environ Microbiol.

[CR23] Skawiński P (2010). Tourism management in the Tatra National Park. Folia Turistica–Tourism and Ecology.

[CR24] Smoroń S, Kowalczyk A (2008). Surface water quality in the tourist areas of the western Carpathians part 3. The analysis of causative factors affecting the aquatic environment in communes of the upper Dunajec catchment basin. Water-Environment-Rural Areas.

[CR25] Smoroń S, Twardy S (2003). The impact of variable intensity of tourist-recreational movement on the river water quality of the Biały and Czarny Dunajec. Water-Environment-Rural Areas.

[CR26] Smoroń S, Twardy S (2006). Concentrations and loads of N-NO_3_, N-NH_4_, PO_4_ and BOD_5_ in waters of the upper Dunajec (in the years 1985-1998). J Water Land Dev.

[CR27] Smoroń S, Twardy S, Janota D (2007). Surface water quality in tourist areas of the Western Carpathians. Part 2. The concentration of business activities connected with tourism services. Water-Environment-Rural Areas.

[CR28] St Laurent J, Mazumder A (2012) The influence of land-use composition on fecal contamination of riverine source water in southern British Columbia. Water Resour Res 48(12). doi:10.1029/2012WR012455

[CR29] Stutter MI, Lumsdon DG (2008). Interactions of land use and dynamic river conditions on sorption equilibria between benthic sediments and river soluble reactive phosphorus concentrations. Water Res.

[CR30] Szalińska E, Dominik J (2006). Water quality changes in the upper Dunajec watershed, Southern Poland. Pol J Environ Stud.

[CR31] Tong S, Chen W (2002). Modeling the relationship between land use and surface water quality. J Environ Manag.

[CR32] Tye AM, Rawlins BG, Rushton JC, Price R (2016). Understanding the controls on sediment-P interactions and dynamics along a non-tidal river system in a rural–urban catchment: the River Nene. Appl Geochem.

[CR33] Walker FR, Stedinger JR (1999). Fate and transport model of Cryptosporidium. J Environ Eng.

[CR34] Whitehead PG, Lack T (1982) Dispersion and self-purification of pollutants in surface water systems. A report by IHP working group 6.1. UNESCO Technical papers in hydrology

